# Effectiveness of the GI Genius Computer-Aided Detection System Versus Standard Colonoscopy: A Systematic Review and Meta-Analysis of Randomized Controlled Trials

**DOI:** 10.7759/cureus.94624

**Published:** 2025-10-15

**Authors:** Aliya Sattar, Arifa Sattar, Muhammad Haris Khan, Maheen Zahid, Simahir Tariq, Neha Choudhary, Muneeba Shaukat, Shermeen Usman, Shakeeba Zubair, Yeman Ahmed, Sarah Aijaz

**Affiliations:** 1 Internal Medicine, Capital Hospital CDA, Islamabad, PAK; 2 Internal Medicine, Services Institute of Medical Sciences, Lahore, PAK; 3 Accident and Emergency, Medical Teaching Institution (MTI) District Headquarter (DHQ) Teaching Hospital, Dera Ismail Khan, PAK; 4 Medicine, King Edward Medical University, Lahore, PAK; 5 Internal Medicine, Rashid Latif Medical College, Lahore, PAK; 6 Medicine, Karachi Medical and Dental College, Karachi, PAK; 7 General Medicine, Kaliganj Diagnostic and Hospital, Gazipur, BGD; 8 Internal Medicine, United Medical & Dental College, Karachi, PAK

**Keywords:** adenoma detection rate, colonoscopy, computer-aided detection, gi genius, meta-analysis, randomized trials

## Abstract

Colorectal cancer prevention relies on high-quality colonoscopy, yet clinically relevant lesions are still missed. GI Genius is an FDA-cleared computer-aided detection (CADe) system that flags suspected polyps in real time. We conducted a Preferred Reporting Items for Systematic reviews and Meta-Analyses (PRISMA)-aligned systematic review and meta-analysis of randomized controlled trials in adults comparing GI Genius-assisted versus standard colonoscopy. PubMed, Ovid Embase, and Cochrane Central Register of Controlled Trials (CENTRAL) were searched from the earliest available records in each database through August 25, 2025, with citation chasing. Seven trials (n = 9,639) met the eligibility criteria. Random-effects meta-analyses showed that GI Genius increased the adenoma detection rate (ADR) compared with standard colonoscopy (RR 1.12, 95% CI 1.03-1.22; I²=64%). Secondary outcomes also favored GI Genius: polyp detection rate (PDR; two trials, n = 5,225; RR 1.05, 95% CI 1.01-1.10; I² = 0%), sessile serrated lesion detection rate (SSLDR; four trials, n = 7,013; RR 1.27, 95% CI 1.11-1.47; I² = 11%), and lesion counts (adenomas per colonoscopy/mean adenomas per patient, six trials, n = 9,253; standardized mean difference (SMD) 0.15, 95% CI 0.09-0.20; I² = 29%; polyps per colonoscopy/mean polyps, two trials, n = 5,228; SMD 0.10, 95% CI 0.05-0.15; I² = 0%). Advanced ADR (three trials, n=6,328) showed no significant difference (RR 1.01, 95% CI 0.90-1.13; I² = 6%). Safety reporting was limited and was not included in the meta-analysis. In summary, adjunctive GI Genius improves clinically meaningful detection, increasing ADR, PDR, SSLDR, and per-procedure lesion counts, without a detectable effect on advanced adenoma detection. These findings support routine activation of GI Genius during eligible colonoscopies, contingent on appropriate team training and consistent response to system prompts. Larger multicenter trials with standardized methods and long-term clinical endpoints are warranted.

## Introduction and background

Colorectal cancer (CRC) is a major global health burden and the third most commonly diagnosed cancer [1,2]. It is also the second leading cause of cancer-related death, with approximately 1.9 million new cases and 930,000 deaths annually [1,2]. Prevention relies on detecting and removing precancerous adenomas [3]. Colonoscopy remains central to CRC prevention, as detection and immediate polypectomy reduce both CRC incidence and mortality [3,4]. The adenoma detection rate (ADR) is a key quality indicator, inversely associated with interval CRC and CRC mortality [5,6]. However, colonoscopy is imperfect: tandem-colonoscopy studies suggest adenoma miss rates of approximately 20-26%, particularly for small, flat, and serrated lesions, with operator and procedural factors contributing to variability [7].

AI-based computer-aided detection (CADe) systems analyze colonoscopy video in real time and alert endoscopists to subtle mucosal abnormalities. Randomized trials and meta-analyses indicate that CADe can increase ADR, particularly for diminutive and flat polyps [8,9]. Among available systems, GI Genius (Medtronic plc, Dublin, Ireland) was the first CADe cleared by the US FDA via the de novo pathway in 2021; it integrates with standard endoscopy stacks and provides on-screen visual cues and audio alerts [10]. Pragmatic randomized data also report gains in per-procedure detection metrics alongside increased ADR in routine practice [11]. Expert reviews note that these improvements are concentrated in subtle, non-advanced lesions and that effect sizes may vary depending on study design and operator factors [10,12].

Despite growing interest in CADe, the device-specific impact of GI Genius on key detection outcomes remains incompletely quantified, particularly whether benefits extend beyond ADR to serrated lesions and per-procedure lesion yield across diverse settings. To address this gap, we performed a Preferred Reporting Items for Systematic reviews and Meta-Analyses (PRISMA)-aligned meta-analysis of randomized trials comparing GI Genius-assisted with standard colonoscopy, providing pooled, device-specific effect estimates for outcomes including ADR, polyp detection rate (PDR), advanced ADR (AADR), sessile serrated lesion detection rate (SSLDR), and lesion counts per procedure, such as adenomas per colonoscopy/mean adenomas per patient (APC/MAP) and polyps per colonoscopy/mean polyps (PPC/MP).

## Review

Methodology 

Eligibility Criteria

We included randomized controlled trials (RCTs; parallel or crossover) enrolling adults (≥18 years) undergoing colonoscopy for any clinical indication. The intervention was real-time CADe using the GI Genius system (Medtronic plc/Cosmo Pharmaceuticals NV, Dublin, Ireland), and the comparator was standard colonoscopy without AI. Trials were required to report at least one detection outcome. We excluded pediatric populations, non-colonoscopy procedures, AI systems other than GI Genius (including head-to-head AI comparisons), non-randomized or quasi-experimental designs, technical-only reports, abstracts without peer-reviewed full text, narrative reviews, and duplicates (retaining the most complete or most recent report). No date limits were applied; studies had to be in English or have an English translation.

Information Sources and Search Strategy

We searched PubMed, Ovid Embase, and the Cochrane Central Register of Controlled Trials (CENTRAL) from inception to August 25, 2025, supplemented by backward and forward citation chasing of included studies and relevant reviews. For PubMed, we used a three-block Boolean strategy with OR within blocks and AND between blocks, employing MeSH terms and [tiab] fields with truncation where appropriate, without additional limits. Block 1 (AI/CADe) included terms such as “artificial intelligence,” “computer-aided detection” (CADe/CADx), “machine learning,” “computer vision,” and MeSH terms “Pattern Recognition, Automated” and “Image Processing, Computer-Assisted.” Block 2 (colonoscopy) included “Colonoscopy”[MeSH] or colonoscop*[tiab]. Block 3 (lesion/outcomes) included “Adenoma”[MeSH], “Polyps”[MeSH], adenoma*, polyp*, neoplas*, “sessile serrated,” “adenoma detection rate” (ADR), “polyp detection rate” (PDR), “missed adenoma*,” and “missed polyp*.” Exact PubMed search strings and the adapted Embase and CENTRAL strategies (including interfaces, run dates, and database-level hit counts) are provided in Appendix A.

Study Selection

Two reviewers independently screened titles and abstracts, followed by full-text assessment against prespecified criteria. Deduplication used title normalization alongside first author, year, DOI, or trial identifier, and journal. Reasons for full-text exclusion were recorded, and disagreements were resolved by consensus. When multiple reports described the same trial, data were extracted from the most complete or most recent report and cross-checked for consistency.

Data Extraction and Outcomes

Using standardized forms, two reviewers independently extracted study characteristics (study design; single- vs. multicenter), population details (indication; special-risk subgroups), intervention and comparator, and outcomes. The primary outcome was ADR (per patient). Secondary outcomes were PDR (per patient), AADR (per study definition), SSLDR (per patient), and lesion counts (APC/MAP and PPC/MP). Rate outcomes were extracted per patient; count outcomes were extracted per procedure. Intention-to-treat (ITT) or modified ITT denominators were used when available.

Risk of Bias 

Two reviewers assessed risk of bias using the Cochrane Risk of Bias 2 (RoB 2) tool (Cochrane Collaboration; version 6.3, updated 2022), with consensus resolution across the five standard domains. Risk-of-bias plots were generated using the robvis web application (developed by Luke A. McGuinness and J.P.T. Higgins).

Statistical Analysis

Meta-analyses were conducted in Review Manager (RevMan) v5.4.1 (Cochrane Collaboration, London, UK) using random-effects models. Dichotomous outcomes (ADR, PDR, AADR, and SSLDR) were pooled as RRs with 95% CIs; continuous outcomes (APC/MAP and PPC/MP) were pooled as standardized mean differences (SMDs) or mean differences with 95% CIs. Percentages were converted to event counts using reported arm denominators. For single-arm zero cells, a 0.5 continuity correction was applied per RevMan; double-zero studies were excluded from risk-ratio pooling per software defaults. Multi-arm trials were combined at the arm level to avoid double-counting, and for crossover designs, first-period data were used. Per-patient and per-polyp metrics were not mixed within any meta-analysis. Between-study heterogeneity was summarized using I². Certainty of evidence for detection outcomes was appraised using GRADE, with reasons for any downgrades reported.

Small-Study Effects/Publication Bias

We prespecified assessment of small-study effects using funnel plots and Egger’s regression when ≥10 studies contributed to an outcome. When fewer than 10 studies were available, funnel plots were not constructed or interpreted, as such tests are underpowered and potentially misleading.

Results 

Study Selection 

**Figure 1:**
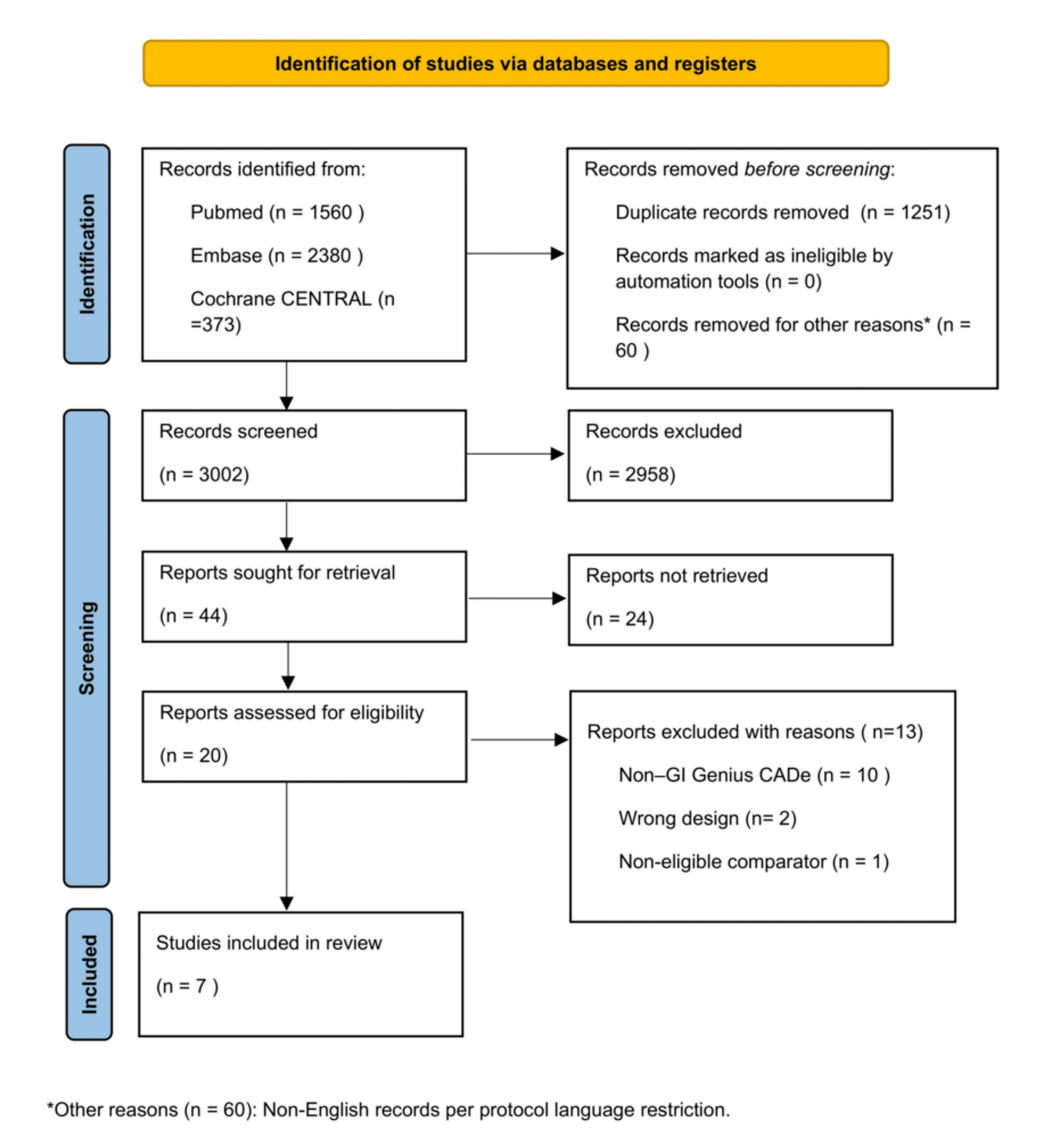
PRISMA flowchart showing study selection process CADe, computer-aided detection; PRISMA, Preferred Reporting Items for Systematic reviews and Meta-Analyses

We identified 4,313 records from PubMed, Embase, and Cochrane CENTRAL. After removing duplicates (n = 1,251) and non-English records (n = 60), 3,002 records were screened, of which 2,958 were excluded. We sought retrieval of 44 reports, but 24 could not be obtained, the majority being protocol-only or abstract-only studies. Twenty full texts were assessed, and 13 were excluded: 10 evaluated non-GI Genius CADe systems, two were non-randomized or secondary analyses, and one used a non-eligible comparator. Ultimately, seven RCTs were included (Figure 1).

Study Characteristics 

The seven included RCTs compared real-time GI Genius CADe with standard colonoscopy and reported at least one detection outcome. Rate outcomes (ADR, PDR, AADR, and SSLDR) were extracted per patient, whereas count outcomes (APC/MAP and PPC/MP) were extracted per procedure. Study characteristics are summarized in Table 1.

**Table 1:** Characteristics of the included studies ^*^ Thiruvengadam et al. (2024) [17]: age was reported as median (IQR); no mean ± SD (years) available NR, not reported; RCT, randomized controlled trial

Study	Country	Design	Center(s)	Sample size (N)	Sex		Age, mean ± SD (years)^*^		Device	Control
					Male, n	Female, n	AI	Control		
Seager et al. (2024) [11]	UK	RCT	Multicenter	2032	1132	900	62.5 ± 10.8	62.2 ± 10.8	GI Genius	Standard colonoscopy
Repici et al. (2020) [13]	Italy	RCT	Multicenter	685	337	348	61.5 ± 9.7	61.1 ± 10.6	GI Genius	Standard colonoscopy
Wallace et al. (2022) [14]	Italy, UK, US	RCT	Multicenter	230	157	73	63.0 ± 8.2	64.6 ± 8.1	GI Genius	Standard colonoscopy
Karsenti et al. (2023) [15]	France	RCT	Single center	2015	979	1036	58.4 ± 11.4	58.4 ± 11.8	GI Genius	Standard colonoscopy
Mangas-Sanjuan et al. (2023) [16]	Spain	RCT	Multicenter	3213	1717	1496	60.7 ± 5.8	60.6 ± 5.7	GI Genius	Standard colonoscopy
Thiruvengadam et al. (2024) [17]	US	RCT	Single center	1100	428	669	NR	NR	GI Genius	Standard colonoscopy
Ortiz et al. (2024) [18]	Spain; Italy; Germany; Belgium	RCT	Multicenter	430	174	256	NR	NR	GI Genius	Standard colonoscopy

Risk of Bias 

**Figure 2:**
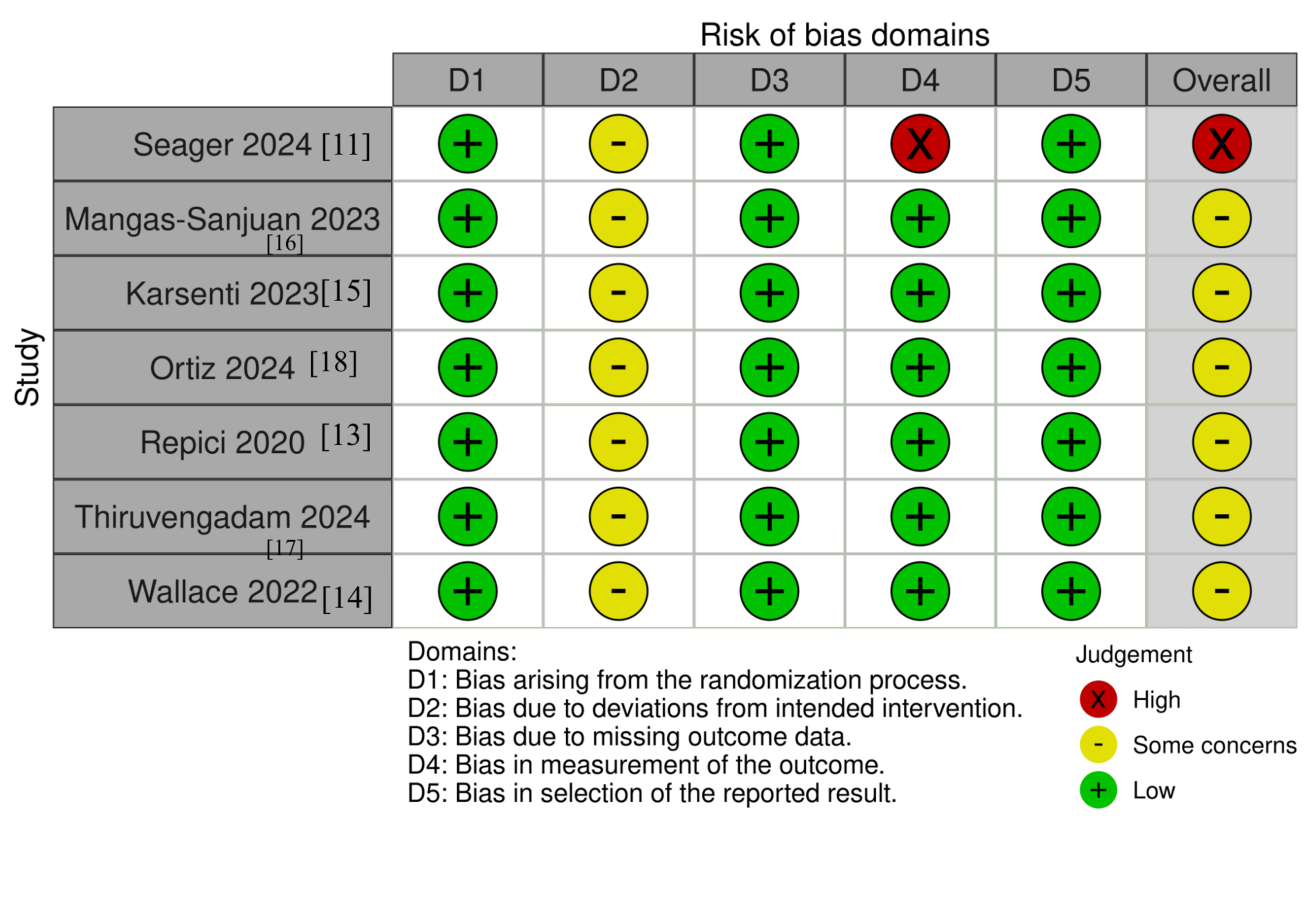
Risk of bias (RoB 2) traffic-light plot of the included randomized trials Domains: D1 = randomization process; D2 = deviations from intended interventions; D3 = missing outcome data; D4 = measurement of the outcome; D5 = selection of the reported result [11,13-18]

**Figure 3:**
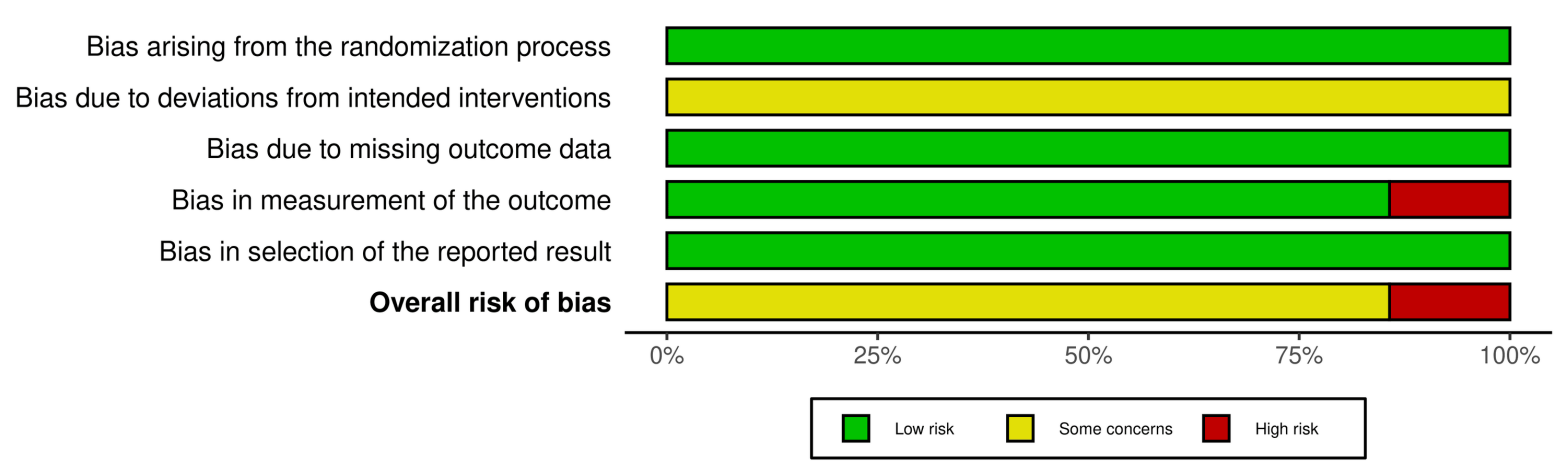
Risk of bias (RoB 2) summary plot Bars indicate the proportion of studies judged as low risk (green), some concerns (yellow), or high risk (red) for each domain and overall.

Risk-of-bias assessment using RoB 2 showed that six of the seven RCTs were rated as low risk or with some concerns, primarily in domain 2 (deviations from intended intervention) due to the impossibility of blinding endoscopists. One study was judged to be at high overall risk of bias (domain 4: measurement of the outcome). Risk-of-bias traffic-light and summary plots are shown in Figure 2 and Figure 3.

Primary Outcome (ADR) 

**Figure 4:**
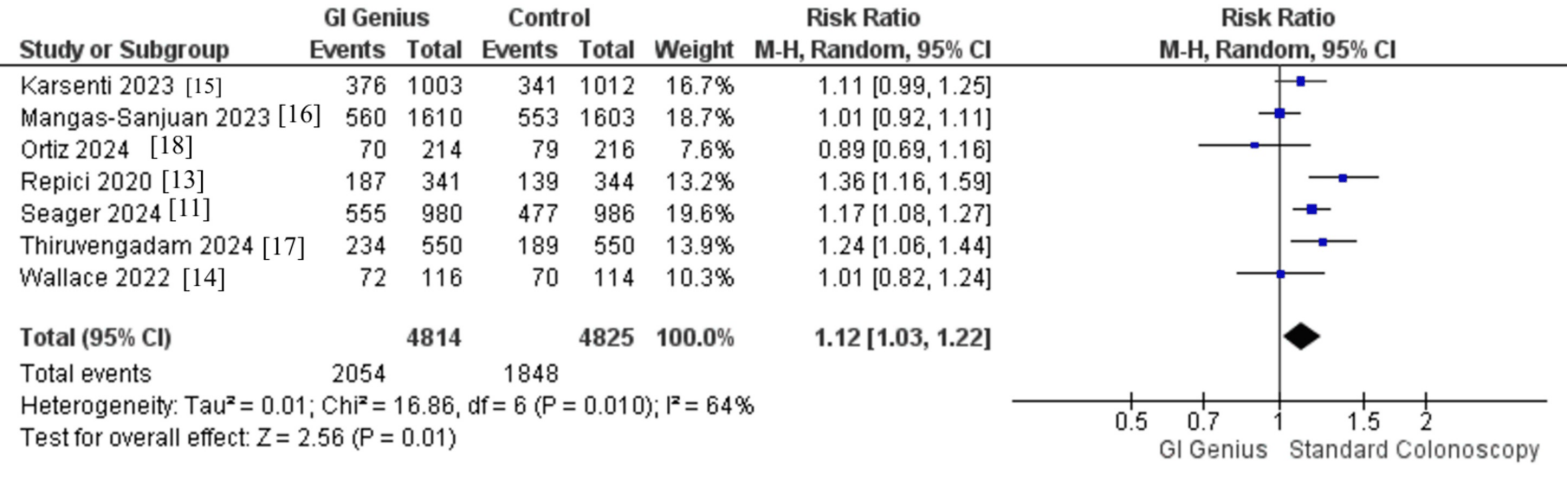
Forest plot comparing ADR: GI Genius vs. standard colonoscopy Pooled effect: RR 1.12 (95% CI 1.03-1.22); I² = 64%; 7 RCTs; n = 9,639 ADR, adenoma detection rate; RCT, randomized controlled trial [11,13-18]

Seven studies, comprising 9,639 patients (GI Genius: 4,814; standard colonoscopy: 4,825), were included in the pooled analysis of ADR. GI Genius significantly improved adenoma detection compared with standard colonoscopy (RR 1.12, 95% CI 1.03-1.22, p = 0.01), representing a 12% relative increase in the likelihood of detecting at least one adenoma. Heterogeneity was moderate (I² = 64%, p = 0.01; τ² = 0.01). Clinically, this implies that for approximately every eight to 10 patients undergoing colonoscopy with GI Genius, one additional adenoma will be detected (Figure 4).

Secondary Outcomes 

**Figure 5:**
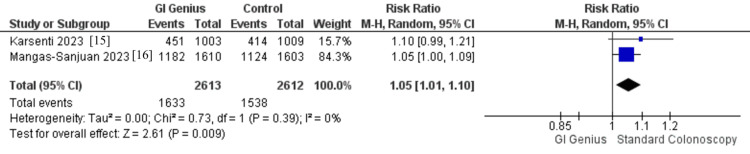
Forest plot comparing PDR: GI Genius vs. standard colonoscopy Pooled effect: RR 1.05 (95% CI 1.01-1.10); I² = 0%; 2 RCTs; n = 5,225 PDR, polyp detection rate; RCT, randomized controlled trial [15,16]

Two studies, including 5,225 patients (GI Genius: 2,613; standard colonoscopy: 2,612), reported PDR. Pooled analysis showed that GI Genius significantly improved PDR compared with standard colonoscopy (RR 1.05, 95% CI 1.01-1.10, p = 0.01), corresponding to a 5% relative increase in the likelihood of detecting at least one polyp. Heterogeneity was absent (I² = 0%, p = 0.38; τ² = 0.00). Clinically, this suggests that roughly 1 in 20 patients undergoing colonoscopy with GI Genius will have an additional polyp detected. While the relative effect is modest, the higher PDR is clinically meaningful, as it captures both adenomas and other lesion types, thereby supporting more comprehensive CRC prevention (Figure 5).

**Figure 6:**
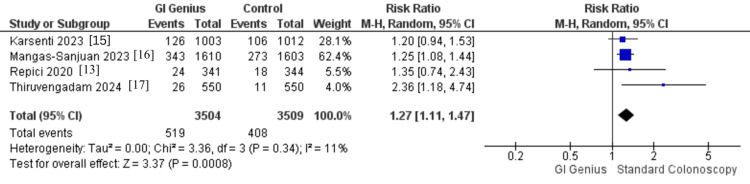
Forest plot comparing SSLDR: GI Genius vs. standard colonoscopy Pooled effect: RR 1.27 (95% CI 1.11-1.47); I² = 11%; 4 RCTs; n = 7,013 RCT, randomized controlled trial; SSLDR, sessile serrated lesion detection rate [13,15-17]

Four RCTs, including 7,013 patients (GI Genius group: 3,504; control group: 3,509), reported SSLDRs. Use of the GI Genius system was associated with a significantly higher SSLDR compared with standard colonoscopy (RR 1.27, 95% CI 1.11-1.47; p = 0.0008). The pooled analysis showed low statistical heterogeneity (I² = 11%, p = 0.34; τ² = 0.00). This represents a 27% relative increase in the likelihood of detecting at least one sessile serrated lesion (SSL) with GI Genius. Clinically, this improvement is meaningful, as SSLs are subtle, flat, and often overlooked, despite being precursors in the serrated pathway to CRC. Enhanced detection with AI assistance may therefore help reduce the risk of interval cancers (Figure 6).

**Figure 7:**
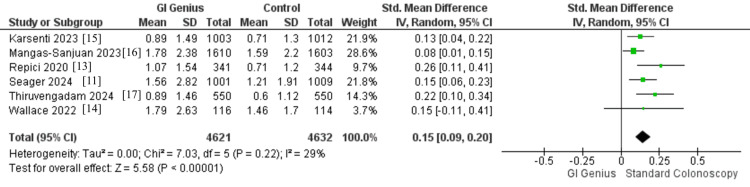
Forest plot comparing APC/MAP Pooled effect: SMD 0.15 (95% CI 0.09-0.20); I² = 29%; 6 RCTs; n = 9,253 APC/MAP, adenomas per colonoscopy/mean adenomas per patient; RCT, randomized controlled trial; SMD, standardized mean difference [11,13-17]

A total of six RCTs, comprising 9,253 participants (4,621 in the GI Genius group and 4,632 in the standard colonoscopy group), reported data on APC or MAP. Pooled analysis demonstrated a statistically significant improvement in adenoma detection with GI Genius compared with standard colonoscopy (SMD = 0.15, 95% CI: 0.09-0.20, p < 0.00001). Heterogeneity across studies was low (I² = 29%, p = 0.22; τ² = 0.00), suggesting consistent findings among trials. These results indicate that AI-assisted colonoscopy modestly but robustly increases the average number of adenomas detected per procedure. Clinically, even small incremental gains in adenoma detection are valuable, as each additional lesion identified and removed may reduce the long-term risk of CRC (Figure 7).

**Figure 8:**
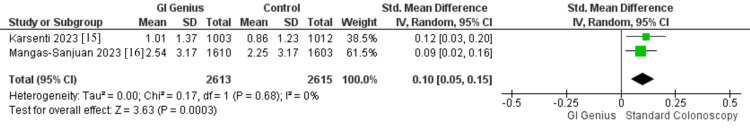
Forest plot comparing PPC/MP Pooled effect: SMD 0.10 (95% CI 0.05-0.15); I² = 0%; 2 RCTs; n = 5,228 PPC/MP, polyps per colonoscopy/mean polyps; RCT, randomized controlled trial; SMD, standardized mean difference [15,16]

Two RCTs, including 5,228 participants (2,613 in the GI Genius group and 2,615 in the control group), assessed the number of polyps detected per colonoscopy. Pooled analysis demonstrated a significant improvement with GI Genius compared with standard colonoscopy (SMD = 0.10, 95% CI: 0.05-0.15, p = 0.0003). No heterogeneity was observed between studies (I² = 0%, p = 0.68). This indicates that GI Genius increases the detection of all polyps, including adenomas, hyperplastic polyps, and SSLs. Clinically, this broader detection capacity may enhance preventive effectiveness by ensuring removal of a wider range of lesions that could contribute to colorectal neoplasia (Figure 8).

**Figure 9:**
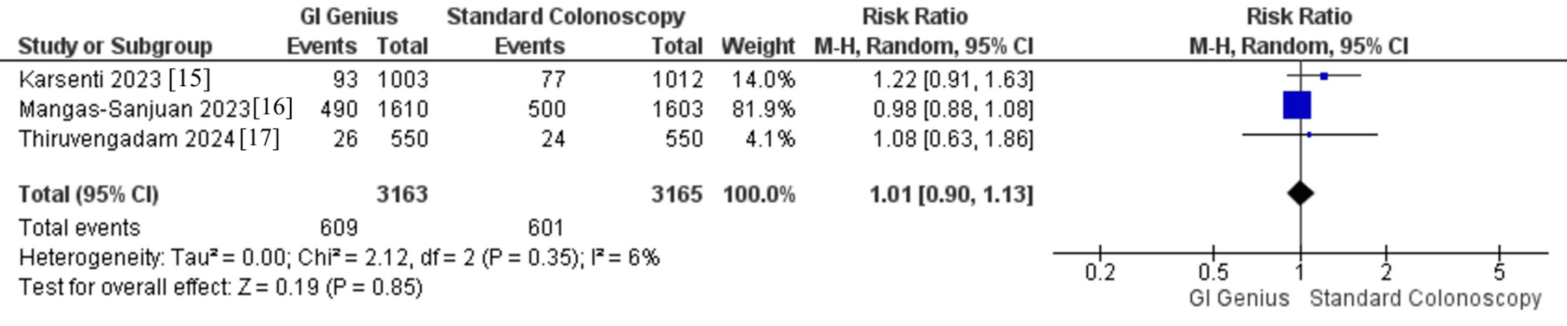
Forest plot comparing AADR: GI Genius vs. standard colonoscopy Pooled effect: RR 1.01 (95% CI 0.90-1.13); I² = 6%; 3 RCTs; n = 6,328 AADR, advanced adenoma detection rate; RCT, randomized controlled trial [15-17]

Three studies, including 6,328 patients (3,163 in the GI Genius group and 3,165 in the standard colonoscopy group), assessed AADR. The pooled analysis showed no significant difference between GI Genius and standard colonoscopy (RR: 1.01, 95% CI: 0.90-1.13, p = 0.85). Heterogeneity was low (I² = 6%, p = 0.35; τ² = 0.00). Clinically, this indicates that while GI Genius enhances overall adenoma and polyp detection, it does not appear to substantially improve detection of advanced adenomas, the most clinically relevant precursors of CRC. Therefore, the benefits of GI Genius may be primarily related to improved recognition of smaller or less advanced lesions (Figure 9).

Certainty of Evidence (GRADE) 

Certainty was moderate for ADR (downgraded for inconsistency) and AADR (downgraded for imprecision) and high for PDR, SSLDR, APC/MAP, and PPC/MP (Table 2). Collectively, these findings indicate that AI-assisted colonoscopy consistently improves the detection of adenomas, polyps, and SSLs, with the strongest effects observed for ADR, SSLDR, APC, and PPC, while the impact on advanced adenomas remains uncertain.

**Table 2:** Summary of findings (GRADE): GI Genius vs. standard colonoscopy Effect estimates are presented with 95% CIs and certainty ratings. AADR, advanced adenoma detection rate; ADR, adenoma detection rate; APC, adenomas per colonoscopy; MAP, mean adenomas per patient; MP, mean polyps; PDR, polyp detection rate; PPC, polyps per colonoscopy; SMD, standardized mean difference; SSLDR, sessile serrated lesion detection rate

Outcome	No. of studies (patients)	Risk of bias	Inconsistency	Indirectness	Imprecision	Other considerations	Relative effect (95% CI)	Certainty
ADR [11,13-18]	Seven RCTs (9,639)	Not serious	Serious (I² = 64%)	Not serious	Not serious	None	RR 1.12 (1.03-1.22)	Moderate
PDR [15,16]	Two RCTs (5,225)	Not serious	Not serious (I² = 0%)	Not serious	Not serious	None	RR 1.05 (1.01-1.10)	High
AADR [15-17]	Three RCTs (6,328)	Not serious	Not serious (I² = 6%)	Not serious	Serious (wide CI, crosses no effect)	None	RR 1.01 (0.90-1.13)	Moderate
SSLDR [13,15-17]	Four RCTs (7,013)	Not serious	Not serious (I² = 11%)	Not serious	Not serious	None	RR 1.27 (1.11-1.47)	High
APC/MAP [11,13-17]	Six RCTs (9,253)	Not serious	Not serious (I² = 29%)	Not serious	Not serious	None	SMD 0.15 (0.09-0.20)	High
PPC/MP [15,16]	Two RCTs (5,228)	Not serious	Not serious (I² = 0%)	Not serious	Not serious	None	SMD 0.10 (0.05-0.15)	High

Publication Bias

For all outcomes, fewer than 10 studies contributed (ADR, k = 7; all other outcomes, k ≤ 3). Accordingly, in line with our prespecified plan, we did not generate funnel plots or perform formal tests for small-study effects, as these analyses would be underpowered and potentially misleading.

Discussion 

Principal Findings 

This meta-analysis evaluated whether the GI Genius CADe system enhances colorectal neoplasia detection compared with standard white-light colonoscopy in randomized trials, focusing on core detection outcomes (ADR, PDR, AADR, and SSLDR), lesion counts (APC/MAP and PPC/MP), and methodological appraisal (RoB 2, GRADE).

Across seven RCTs (n = 9,639), GI Genius consistently improved detection compared with standard colonoscopy: ADR increased (RR 1.12, 95% CI 1.03-1.22), PDR increased (RR 1.05, 95% CI 1.01-1.10), and SSLDR increased (RR 1.27, 95% CI 1.11-1.47). Lesion yield was also higher for APC/MAP (SMD 0.15, 95% CI 0.09-0.20) and PPC/MP (SMD 0.10, 95% CI 0.05-0.15). AADR showed no significant difference (RR 1.01, 95% CI 0.90-1.13). RoB 2 assessments indicated “some concerns” in six trials and high risk in one trial (outcome measurement). According to GRADE, certainty was moderate for ADR (downgraded for inconsistency) and AADR (downgraded for imprecision) and high for PDR, SSLDR, APC/MAP, and PPC/MP.

Comparison With Prior Evidence

Previous meta-analyses pooling mixed CADe platforms generally report ADR gains with AI assistance [9,19,20]. By restricting analysis to a single, widely deployed platform (GI Genius), this review isolates device-specific performance [10] and demonstrates a recurring pattern across AI systems: larger benefits for subtle lesions (small adenomas and SSLs) than for advanced adenomas [9,19,20]. This aligns with clinical experience, as advanced lesions are more conspicuous, whereas CADe highlights fleeting, low-contrast abnormalities [9,10,12]. Even modest ADR increases are associated with lower interval CRC incidence and mortality at the population level [5,6].

Clinical Interpretation and Implementation 

These findings support GI Genius as an adjunct to high-definition colonoscopy. Real-world evidence suggests that benefits depend on consistent system activation and endoscopist engagement with prompts; utilization can vary [21,22]. False-positive signals are common but typically brief and manageable, resulting in limited workflow disruption. Combining CADe with mucosal-exposure aids (e.g., distal attachments) may further enhance detection, particularly for right-sided and serrated lesions [23-25].

Strengths and Limitations 

Strengths of this meta-analysis include its exclusive RCT evidence base, prespecified per-patient denominators for rate outcomes, clear unit-of-analysis rules, random-effects synthesis, and GRADE appraisal. Limitations include moderate heterogeneity for ADR (I² = 64%), variation in study design (parallel vs. tandem/crossover), and potential performance bias due to partial blinding, factors known to influence CADe effect estimates [12,26]. Most trials were conducted in Europe or Asia, which may limit generalizability. Small-study effects could not be reliably evaluated because fewer than 10 trials contributed to each outcome.

Implications for Practice and Research 

Where available, routine activation of GI Genius during colonoscopy is reasonable to enhance overall detection, particularly for SSLs, provided that teams are trained to respond efficiently to prompts [19,21]. Future research priorities include large, multicenter RCTs with standardized designs and reporting, evaluation across diverse practice settings, and long-term clinical endpoints. Trials exploring combinatorial quality-improvement strategies (e.g., CADe plus exposure-enhancing tools) and next-generation AI may further optimize detection and workflow [20,24].

## Conclusions

This meta-analysis demonstrates that adjunctive use of the GI Genius CADe system with standard high-definition colonoscopy substantially improves clinically relevant lesion detection. Integration of AI resulted in a significant increase in ADR, the most critical benchmark for colonoscopy quality. GI Genius also markedly improved the detection of SSLs, which are subtle and frequently missed, showing a 27% relative improvement. The system increased overall polyp detection and the mean number of lesions per procedure; however, no measurable effect was observed on advanced adenoma detection. Although small-study effects could not be formally assessed (k < 10), the direction of benefit was consistent across trials, with clear improvement in key quality metrics.

These robust findings support consideration of routine activation of GI Genius CADe during eligible colonoscopies. By adopting this technology and ensuring proper team training to respond effectively to its prompts, endoscopy units can significantly enhance CRC prevention efforts. Future research should continue refining AI implementation across diverse practice settings and investigate its long-term impact on reducing cancer incidence.

## Appendices

Appendix A

Information Sources and Search Strategy

Databases and Platforms

PubMed - searched August 25, 2025, 1560 records

Embase (Ovid) - searched August 25, 2025, 2380 records

Cochrane Central Register - searched August 25, 2025, 373 records

Record counts by database and total are reported in the PRISMA 2020 flow diagram (Figure 1) and corresponding text in the Results section as well.

Coverage: Inception to August 25, 2025.

Limits: English or English translation; humans (applied during screening; not embedded in queries)

Automation tools: None.

Exact Search Strategies

PubMed

("artificial intelligence"[MeSH Terms] OR "artificial intelligence"[tiab] OR

  "computer aided detection"[tiab] OR "computer-aided detection"[tiab] OR CADe[tiab] OR CADx[tiab] OR

  "machine learning"[tiab] OR "deep learning"[tiab] OR

  "convolutional neural network*"[tiab] OR "neural network*"[tiab] OR CNN[tiab] OR

  "computer vision"[tiab] OR

  "pattern recognition, automated"[MeSH Terms] OR "image processing, computer-assisted"[MeSH Terms])

AND

("colonoscopy"[MeSH Terms] OR colonoscop*[tiab])

AND

( "adenoma"[MeSH Terms] OR "polyps"[MeSH Terms] OR

  adenoma*[tiab] OR polyp*[tiab] OR neoplas*[tiab] OR "sessile serrated"[tiab] OR

  "adenoma detection rate"[tiab] OR ADR[tiab] OR

  "polyp detection rate"[tiab] OR PDR[tiab] OR

  "miss rate"[tiab] OR "missed adenoma*"[tiab] OR "missed polyp*"[tiab])

Embase (Ovid; verbatim with line numbers)

1. exp artificial intelligence/

2. artificial intelligence.ti,ab.

3. (computer adj2 aided adj2 detection).ti,ab.

4. CADe.ti,ab.

5. CADx.ti,ab.

6. exp machine learning/

7. machine learning.ti,ab.

8. exp deep learning/

9. deep learning.ti,ab.

10. convolutional neural network.ti,ab.

11. neural network*.ti,ab.

12. CNN.ti,ab.

13. computer vision.ti,ab.

14. 1 OR 2 OR 3 OR 4 OR 5 OR 6 OR 7 OR 8 OR 9 OR 10 OR 11 OR 12 OR 13

15. exp colonoscopy/

16. colonoscop*.ti,ab.

17. 15 OR 16

18. exp adenoma/

19. exp polyp/

20. adenoma*.ti,ab.

21. polyp*.ti,ab.

22. neoplas*.ti,ab.

23. sessile serrated.ti,ab.

24. adenoma detection rate.ti,ab.

25. ADR.ti,ab.

26. polyp detection rate.ti,ab.

27. PDR.ti,ab.

28. miss rate.ti,ab.

29. missed adenoma*.ti,ab.

30. missed polyp*.ti,ab.

31. 18 OR 19 OR 20 OR 21 OR 22 OR 23 OR 24 OR 25 OR 26 OR 27 OR 28 OR 29 OR 30

32. 14 AND 17 AND 31

Cochrane CENTRAL

MeSH descriptor: [Artificial Intelligence] explode all trees

("artificial intelligence" OR "computer aided detection" OR "computer-aided detection"

OR CADe OR CADx OR "machine learning" OR "deep learning"

OR "convolutional neural network" OR "neural network*" OR CNN OR "computer vision"):ti,ab,kw

MeSH descriptor: [Colonoscopy] explode all trees

(colonoscop*):ti,ab,kw

MeSH descriptor: [Adenoma] explode all trees

MeSH descriptor: [Polyps] explode all trees

(adenoma* OR polyp* OR neoplas* OR "sessile serrated" OR

"adenoma detection rate" OR ADR OR

"polyp detection rate" OR PDR OR

"miss rate" OR "missed adenoma*" OR "missed polyp*"):ti,ab,kw

Deduplication and Screening Notes

Deduplication: Manual approach using title normalization, first author, year, DOI/trial identifier, and journal.

Language: Non-English studies were excluded per protocol during screening (count detailed in PRISMA flow).

Screening: Two independent reviewers at title/abstract and full text, with recorded exclusion reasons.
